# Risk of Contralateral Central Compartment Recurrence Following Unilateral Therapeutic Neck Dissection for Papillary Thyroid Carcinoma

**DOI:** 10.1002/jso.70063

**Published:** 2025-08-06

**Authors:** Keren Kaminer, Tal Rozenblat, Itay Shavit, Inbar Finkel, Liat Sasson, Ilan Shimon, Dania Hirsch, Gideon Bachar, Eyal Robenshtok

**Affiliations:** ^1^ Rabin Medical Center, Endocrinology & Metabolism Institute Petach Tikva Israel; ^2^ Faculty of Medicine Tel Aviv University Tel Aviv Israel; ^3^ Department of Otolaryngology, Head and Neck Surgery Rabin Medical Center Petach Tikva Israel; ^4^ Tel Aviv Sourasky Medical Center, Oncology Division Tel Aviv Israel

**Keywords:** central neck dissection, complications, contralateral disease, papillary thyroid carcinoma, prophylactic dissection, recurrence

## Abstract

**Background and Objectives:**

The utility of bilateral central compartment neck dissection (CCND) in patients with papillary thyroid carcinoma (PTC) and unilateral clinically node‐positive disease remains debatable. Previous studies evaluated contralateral occult lymph‐node metastases, which do not necessarily correlate with clinical recurrences. The objective of our study was to evaluate whether unilateral CCND is sufficient, specifically evaluating recurrence in the contralateral central neck.

**Methods:**

Patients with PTC treated with total thyroidectomy and therapeutic unilateral CCND with at least 2 years of follow‐up were included.

**Results:**

A total of 118 patients had unilateral therapeutic CCND, 58% with lateral neck dissection, 63% female, mean age of 48.1 ± 16.3 years. Mean follow‐up was 6.2 ± 3.9 years, tumor size 17.6 ± 12 mm, 39% had minimal extrathyroidal extension (ETE) and 4% had gross ETE. A mean of 2.6 ± 2.6 LN were involved in the central compartment (size 9.4 ± 6.5 mm) and 4.4 ± 4 involved in the lateral neck (size 24.9 ± 14.3 mm). Recurrence on the ipsilateral side was detected in 6 patients (5%), while contralateral central compartment recurrence (the primary outcome) was detected in only 1 patient (1%).

**Conclusions:**

In patients with PTC and unilateral clinically node‐positive central compartment disease, unilateral therapeutic CCND is sufficient, with only 1% risk of recurrence in the contralateral central compartment.

## 1 Introduction

In patients with papillary thyroid carcinoma (PTC), central compartment lymph node (LN) metastases are common, ranging from occult microscopic foci detected in up to 30%–50% of patients on prophylactic dissection, to larger clinically apparent lymph node metastases (“clinical disease”) in up to 20% of patients [1, 2]. As the central compartment is the most common site of LN spread, multiple studies have suggested prophylactic central compartment neck dissection (CCND) in an attempt to decrease locoregional recurrences. However, over time, many centers abandoned this approach due to an increased surgical complication rates and only minor effect on disease outcome, suggesting that microscopic occult metastatic lymph nodes have little impact on the risk of recurrence [3].

When clinically apparent suspicious LN are detected in the central compartment on preoperative ultrasound (US) or during surgery (cN1a disease), there is a clear indication for therapeutic CCND of the affected side. In the presence of unilateral clinically node‐positive central compartment disease, debate exists to this day whether a unilateral CCND is sufficient, given the risk of contralateral central compartment recurrence [4]. Studies on prophylactic bilateral CCND demonstrated contralateral occult disease in 10%–20% of cases [5, 6, 7, 8, 9, 10, 11, 12, 13, 14]. However, there is little published data on the impact of contralateral (uninvolved) CCND on the risk of clinical recurrence, as the presence of occult LNs does not necessarily correlate with structural recurrence risk.

In this study, we evaluate patients who had total thyroidectomy and therapeutic unilateral CCND, with or without lateral dissection, for clinically node‐positive disease detected by imaging or during surgery. The objective of the study is to evaluate the risk of recurrence in the contralateral nonoperated central compartment as the primary outcome.

## 2 Materials and Methods

The electronic records at Rabin Medical Center, a tertiary university‐affiliated medical center, were screened for all patients who were diagnosed with PTC between 2006 and 2022. We included patients with PTC who underwent total thyroidectomy and unilateral CCND. All included patients had unilateral, clinically apparent central compartment lymph node metastases, based on preoperative imaging (US or CT) or intraoperative findings. A minimum of 2 years of follow‐up (up to 2024), including clinical assessment, serum thyroglobulin (Tg) and thyroglobulin antibody (TgAb) levels, and neck US, was required. Patients were excluded if they had prior thyroid or neck surgery, insufficient follow‐up, or if they underwent bilateral central neck dissection.

During the study period, the decision to perform unilateral versus bilateral central neck dissection in patients with clinically node‐positive disease was made at the discretion of the operating surgeon. However, prophylactic contralateral dissection was not routinely performed at our institution, and only patients treated with unilateral therapeutic dissection were included in the study.

The follow‐up period was defined as the time between the thyroidectomy and the last follow‐up or the latest blood tests for TG/TGAb and/or US scan. Recurrence was defined as histologically proven structural recurrence. The study was approved by the Institutional Research Ethics Committee.

The following data were collected for each patient: demographics, medical history, thyroid and neck US scans, fine needle aspiration cytology, serum levels of TSH, Tg, TgAb, surgical reports, histopathology reports, RAI therapy, and surgical complications. Permanent hypoparathyroidism was defined as serum calcium below the normal range at least once more than 1 month after surgery, requiring continued treatment with either calcium supplementation alone or in combination with activated vitamin D for the entire follow‐up period (minimum 2 years). To assess vocal cord mobility, all patients at our institution undergo routine postoperative laryngoscopy on the first day after surgery.

Risk of mortality and risk of disease recurrence were assessed according to the 8th edition of the AJCC/UICC staging system and ATA risk of recurrence stratification.

We extracted data on surgical complications, including vocal cord paralysis and hypoparathyroidism, and recurrence in the central and lateral neck in each case.

### 2.1 Statistical Analysis

All statistical analyses were performed with SPSS v.17.0 (IBM Corp., Armonk, NY, USA). Associations between two categorical variables were examined using *χ* ^2^ test and Fisher's exact test; associations between continuous and quantitative variables were examined using the Mann–Whitney nonparametric *U* test; correlations between binary variables and continuous variables were examined using Pearson's correlation coefficient. A two‐sided *p* value of < 0.05 was considered statistically significant for all analyses.

## 3 Results

### 3.1 Baseline Characteristics

The study cohort consisted of 118 patients who had total thyroidectomy and unilateral CCND for PTC. The mean follow‐up period was 6.2 ± 3.9 years (median—5 years). The mean age at surgery was 48.1 ± 16.3 years (range 18–89 years), and 63% were females (Table 1).

**Table 1:** Baseline characteristics.

	Patients, *n* = 118
Age at surgery, years (mean + SD)	48.1 ± 16.3
Female gender, *n* (%)	75 (63%)
Mean follow‐up, years (mean + SD)	6.2 ± 3.9
Lateral neck dissection, *n* (%)	68 (58%)
Tumor size, mm (mean + SD)	17.6 ± 12
Extrathyroidal extension minimal, *n* (%)	46 (39%)
Extrathyroidal extension gross, *n* (%)	5 (4%)
Multifocal, *n* (%)	79 (66%)
Tall‐cell variant, *n* (%)	9 (8%)
No. of involved LN—central neck (mean ± SD)	2.6 ± 2.6
Maximal LN size—central neck, mm (mean + SD)	9.4 ± 6.5
No. of involved LN—lateral neck (mean + SD)	4.4 ± 4
Maximal LN size—lateral neck (mm), mean + SD	24.9 + 14.3
Extranodal extension (central + lateral), *n* (%)	31 (26%)
I131, *n* (%)	107 (91%)
I131, mCi (mean + SD)	172.4 + 107.1
External beam radiation therapy, *n* (%)	7 (6%)

Abbreviations: CND, central neck dissection; LN, lymph nodes.

Mean tumor size was 17.6 ± 12 mm, 39% had minimal extrathyroidal extension (ETE), and 4% had gross ETE. Histologic variants of PTC included classic (59%), follicular variant (15%), tall cell (8%), and other variants in 18% (oncocytic, unknown). All patients had CCND, with a mean LN yield of 5.6 ± 3.9 (median 4, IQR 3–8), and a mean of 2.6 ± 2.6 metastatic nodes (median 2, IQR 1–4), and a mean LN ratio of 0.65 ± 0.31. The mean size of the metastatic LNs was 9.4 + 6.5 mm. In total, 58% of patients also had lateral compartment neck dissection, with a mean of 4.4 ± 4 involved LN and a mean size of 24.9 ± 14.3 mm. Extranodal extension was detected in 26% of cases. According to the AJCC/UICC 8th edition staging 66% were classified as Stage I, 25% as Stage II, 4% as Stage III, 4% as Stage IV, and 1% undetermined.

A total of 107 patients (91%) were treated with radioiodine (RAI)—88 patients (82%) were treated once, 15 patients (14%) were treated twice, 2 patients (2%) were treated with three courses and 2 patients (2%) were treated with four courses of RAI. Eleven patients (9%) were not treated with RAI due to the following reasons: five had ATA low‐risk disease (≤ 5 involved lymph nodes, all ≤ 2 mm in size); one had concomitant metastatic lung cancer; one had preexisting salivary gland disease; and four refused treatment—one due to advanced age (84 years old), one with major depression, and two due to concerns over side effects. Three patients (3%) were treated with tyrosine kinase inhibitors for metastatic disease.

### 3.2 Recurrence Rates

Contralateral central compartment recurrence (the primary outcome) was detected in only one patient (1%) (Table 2). The patient was treated with total thyroidectomy and ipsilateral CCND and 150 mCi of RAI for left multifocal PTC up to 2.2 cm and 8 of 15 involved LN up to 1.7 cm. Postoperative US was normal, with persistent biochemical disease. Eighteen months after surgery, a contralateral level VI/VII LN was detected on both US and FDG PET/CT just below the clavicle bone. She was treated with Rt CCND as well as Rt level VII (mediastinal) dissection. She was followed for 12 years with no additional events and then was lost to follow‐up.

**Table 2:** Characteristics of seven patients who had therapeutic unilateral CND with central neck recurrence during follow‐up.

Patient no.	Age at surgery (years)	Sex	Lateral neck dissection	Tumor size (mm)	Minimal ETE	Gross ETE	Multifocal disease	I131 cumulative activity (mCi)	Recurrence location	Time to recurrence (months)	Involved lymph nodes—ratio and size (mm)
1	36	F	Y	ND	Y	N	ND	350	Contralateral^a^ (+lateral)	17	Level VI/VII—1/1 13 mm Lateral—4/8 up to 17 mm
2	31	F	N	30	Y	N	Y	270	Ipsilateral (+lateral)	24	Level VI + lateral—11/26 up to 28 mm
3	46	M	Y	14	Y	N	Y	180	Ipsilateral	13	Level VI and VII—3/3 up to 16 mm
4	48	F	Y	25	Y	N	Y	375	Ipsilateral (+lateral)	9	Level VI + lateral—3/8 up to 35 mm
5	57	M	N	30	Y	N	Y	100	Ipsilateral	10	Level VI—1/1, no data on size
6	54	F	Y	7	N	N	Y	150	Ipsilateral	27	Level VI—6/14 up to 18 mm
7	19	M	N	20	N	N	N	150	Ipsilateral (+lateral)	115	Level VI—3/3 up to 9 mm Lateral—3/10 up to 22 mm

Abbreviation: ETE, extrathyroidal extension.

a The only contralateral central neck recurrence.

Ipsilateral central compartment recurrence was more common, and was detected in 6 patients (5%), and in the lateral neck in 12 patients (10%). The median time to recurrence was 18.5 ± 37.6 months (range 9–116 months). The mean number of involved LN was 4.3 ± 2.9 (range 1–10) with mean maximal nodal size of 18.2 ± 8.9 mm (range 9–35 mm). Three of these patients (50%) were treated with one course of RAI and another 3 patients (50%) were treated twice, with an average total dose of 204.1 ± 92 mCi (range 100–375 mCi). The median follow‐up period was 101 ± 46.5 months (range 55–201). In patients with recurrence in the lateral neck, the mean number of involved LN was 3.9 ± 3.1 (range 1–11) and patients were treated with a total mean dose of radioiodine ablation of 331 ± 154 mCi (range 150–745 mCi). Four out of 12 patients had central and lateral dissection and the rest had only lateral neck dissection.

There was no significant correlation between the LN yield or LN ratio from the initial surgery and the risk of central neck recurrence (*p* = 0.17 and 0.18, respectively).

### 3.3 Complications Rate

Vocal cord paralysis was detected in 1 (1%) patient, and permanent hypoparathyroidism was detected in 4 patients (3%). All cases of hypoparathyroidism were biochemically stable and managed with calcium supplementation alone (≤ 1 g/day) in two patients, and activated vitamin D (alphacalcidol ≤ 1 mcg/day) with calcium (≤ 1 g/day) in two patients, consistent with mild disease.

## 4 Discussion

The optimal extent of central neck dissection in patients with PTC remains an area of ongoing debate [4, 5, 6, 7, 11, 15, 16, 17, 18]. Central compartment dissection is typically categorized as therapeutic or prophylactic, depending on whether suspicious lymph nodes (LNs) are identified on preoperative imaging or intraoperatively. While there is broad agreement that clinically involved LNs should be removed, the role of prophylactic dissection, particularly of the contralateral central compartment in patients with unilateral clinically node‐positive disease, is less clear. Specifically, it is uncertain whether removing occult disease from the contralateral clinically uninvolved side provides a meaningful reduction in recurrence risk. In order to evaluate the recurrence rate on the contralateral side in these patients, we conducted a study of 118 patients who underwent TT and unilateral CCND for PTC in our institute, and were followed for 6.2 ± 3.9 years. Our results demonstrated a very low contralateral recurrence rate, with only one patient (1%) requiring dissection of contralateral level VI/VII due to a metastasis just below the clavicle bone.

Multiple previous studies demonstrated that occult micro‐metastases are common in patients with clinically node negative (cN0) PTC, with reported prevalence of 30%–60%, depending on the tumor size [1]. Therefore, many studies suggested routine prophylactic dissection to reduce the risk of recurrence. According to the the 2015 American Thyroid Association (ATA) guidelines, prophylactic CCND should be considered in patients with PTC with clinically uninvolved central compartment lymph nodes (cN0) who have advanced primary tumors (T3 or T4) or clinically involved lateral neck nodes (cN1b), or if the information will be used to plan further steps in therapy [19].

Previous studies evaluating patients with bilateral CND have shown that the presence of ipsilateral central compartment LN metastases is an independent risk factor for contralateral occult disease, including two meta‐analyses [5, 8, 10, 12, 13, 14, 15, 16, 17, 20, 21, 22, 23]. The first was published in 2018, evaluating occult contralateral central compartment LN metastases in patients with unilateral PTC with no evidence of LN on imaging (cN0 disease) [24]. In 6 studies including 1399 patients, the risk of contralateral occult LN ranged from 3.9% to 30.6%, with an average of 11.6%. The presence of occult ipsilateral central LN metastases was a significant risk factor for contralateral occult metastases, with an odds ratio of 12.3. A more recent meta‐analysis published in 2021 evaluated specifically the predictive value of ipsilateral central LN metastasis for contralateral central LN metastasis [11]. Sixteen studies including 3242 patients were included, with an odds ratio of ipsilateral metastasis predicting contralateral central lymph node metastasis of 12.9. Given these high numbers, some authors recommended using frozen biopsy of ipsilateral LN during surgery to evaluate the need for contralateral dissection [11, 21]. However, as our study shows, despite the high prevalence of occult contralateral metastatic LN, the actual risk of recurrence in the contralateral compartment is low, and frozen section of the ipsilateral side does not seem to be indicated.

The rate of central compartment recurrences following prophylactic unilateral versus bilateral CCND was assessed in a meta‐analysis published in 2019 [25]. Out of 25 included studies, 6 compared TT with unilateral CCND versus TT alone, and 10 compared TT with bilateral CCND versus TT alone. While recurrence rates were similar between TT with unilateral CCND and TT alone (8.4% vs. 4.3%, nonsignificant), patients who had TT with bilateral CCND had significantly lower rate of central compartment recurrence (7.8% vs. 3.3%, *p* < 0.00001). Importantly, there were no data on the recurrence pattern (side of recurrence) in the included studies. Given these results, the authors concluded prophylactic bilateral CCND is required in patients with PTC > 1 cm. This study, like many other studies, draws conclusions regarding the extent of surgery, without adequate data on the pattern of recurrence and whether the recurrence occurred on the side of the tumor or the contralateral side. As our study demonstrates, contralateral neck dissection can be advocated only if recurrence actually occurs on that side, and should not be based on the total number of central compartment recurrences.

Local recurrence rates are reported in up to 9% of patients in clinically N0 patients undergoing pCCND, however, several studies demonstrated that most recurrences are in the lateral compartment (Levels III–IV) [26]. Lang et al. [27] studied 341 patients who underwent TT and unilateral pCCND, with a very low incidence of locoregional recurrence, with a 5‐year recurrence rate of 5.1% and a 10‐year recurrence rate of 6.1%. Most of the recurrences were located in the lateral compartment. The higher rate of recurrences in the lateral compartment is consistent with our results which demonstrated that among patients in the unilateral CCND group, central compartment recurrence in the ipsilateral side was 5% and recurrence in the lateral neck was detected in 10%.

Lymph node yield and LN ratio have been previously shown to correlate with recurrence and adequacy of surgery in patients with PTC. A low LN yield may reflect insufficient clearance of the central compartment, while a high LN ratio likely reflects a greater extent of nodal involvement. Several studies demonstrated that a lower central lymph node yield and higher lymph node ratio are associated with increased risk of persistent or recurrent disease [28, 29]. Yang and colleagues found a central neck LN yield of < 4 as an independent predictor of recurrence in unilateral dissection, while Yu and colleagues suggested a threshold of < 11 for bilateral dissection [28, 30]. Schneider and colleagues found LN ratio to be a better predictor of recurrence than absolute node count, with ratio of ≥ 0.7 predicting worse disease‐free survival [31]. In our cohort, the mean central LN yield was 5.6 and the mean LN ratio was 0.65, both within the ranges considered adequate in these prior studies. Importantly, we found no significant association between either parameter and the risk of central neck recurrence, probably due to insufficient sample size for this analysis. Overall, these results suggest that the extent of unilateral therapeutic CCND performed in our study was oncologically adequate

The rate of surgical complications in our study was low, with 1% vocal cord paralysis and 3% permanent hypoparathyroidism. Unlu et al. [32] conducted a retrospective study with 186 patients evaluating the rate of complications among patients undergoing CCND (unilateral CCND ± lateral neck dissection or bilateral CCND ± lateral neck dissection) compared to TT without CCND, and found that CCND alone was an independent risk factor for increased both total and transient hypoparathyroidism, however, the difference was not significant between the unilateral versus bilateral CCND group. Also, the rate of permanent vocal cord paralysis was significantly higher among patients undergoing TT and CCND compared to patients undergoing TT. Giordano et al. [33] conducted a retrospective study of 1087 patients with N0 PTC. Patients were divided into three groups—total thyroidectomy, TT with ipsilateral CCND, and TT with bilateral CCND. There was no significant difference between the three groups in the rate of transient and permanent vocal cord paralysis (0.5% in the unilateral CCND vs. 2.3% in the bilateral CCND for permanent VCP, *p* = 0.099). However, there was a higher rate of permanent hypoparathyroidism in patients undergoing bilateral CCND (7% in the unilateral CCND vs. 16.2% in the bilateral CCND for permanent VCP, *p* < 0.001). In our study, permanent vocal cord paralysis was detected in 1 (1%) patient, and permanent hypoparathyroidism was detected in 4 patients (3%).

Only one previous study evaluated the risk of contralateral central compartment recurrence in patients with unilateral node‐positive disease [18], but unlike our study, all patients had bilateral CCND (therapeutic in the ipsilateral side and prophylactic on the contralateral side). The risk of recurrence in the contralateral central compartment was 1.2% in patients who had no contralateral LN on initial pathology, and 0.8% in patients who had contralateral LN on pathology. Thus, our study is unique in that it evaluates the real‐world risk of contralateral central neck recurrence without prophylactic dissection, directly addressing the clinical relevance of unilateral versus bilateral central neck dissection.

A key strength of our study lies in its unique design, which directly addresses the practical question of whether unilateral CCND is oncologically sufficient for patients with clinically node‐positive PTC. Unlike previous studies that focused on detecting occult contralateral LN metastases through prophylactic CCND, an approach that does not necessarily predict clinical recurrence, our study evaluates the actual risk of contralateral central compartment recurrence. By using a compartment‐specific recurrence rate as our primary outcome and with a sufficient follow‐up period, our findings provide clinically relevant and practical data to guide surgical decision‐making.

Our study has several limitations. The study was retrospective, which may have limited the accuracy and completeness of the collected data. Second, here was some heterogeneity in pathological reports quality and details over the years. Moreover, the procedure was performed over the last two decades by different surgeons using different techniques, which may have influenced the recurrence rate and rate of complications.

## 5 Conclusions

In patients with PTC and ipsilateral node‐positive disease detected on imaging or during surgery, unilateral therapeutic CCND is effective, with < 1% risk of recurrence in the contralateral (nonoperated) central compartment, and associated with low complication rates. Bilateral CCND in this setting is unlikely to add clinical benefit while increasing the risk of complications.

## Disclosure

The authors have nothing to report.

## Synopsis

In patients with papillary thyroid carcinoma (PTC) and clinically node‐positive disease in the central neck, the extent of dissection remains a matter of debate. In this retrospective study of 118 patients with unilateral central neck involvement identified on ultrasound or intra‐operatively, we found that unilateral dissection was sufficient, with contralateral recurrence occurring in only 1%. These findings suggest that unilateral central neck dissection is oncologically sufficient in such cases, avoiding unnecessary bilateral surgery and its associated risks.

## Data Availability

The data that support the findings of this study are available on request from the corresponding author. The data are not publicly available due to privacy or ethical restrictions.
